# Case Report: The impact of severe cryptosporidiosis on the gut microbiota of a pediatric patient with CD40L immunodeficiency

**DOI:** 10.3389/fcimb.2023.1281440

**Published:** 2023-10-26

**Authors:** Antonia Piazzesi, Stefania Pane, Alessandra Russo, Federica Del Chierico, Paola Francalanci, Nicola Cotugno, Paolo Rossi, Franco Locatelli, Paolo Palma, Lorenza Putignani

**Affiliations:** ^1^ Unit of Human Microbiome, Bambino Gesù Children’s Hospital, IRCCS, Rome, Italy; ^2^ Unit of Microbiomics, Bambino Gesù Children’s Hospital, IRCCS, Rome, Italy; ^3^ Department of Pathology, Bambino Gesù Children’s Hospital, IRCCS, Rome, Italy; ^4^ Unit of Clinical Immunology and Vaccinology, Bambino Gesù Children’s Hospital, IRCCS, Rome, Italy; ^5^ Department of Systems Medicine, Chair of Pediatrics, University of Rome “Tor Vergata”, Rome, Italy; ^6^ Academic Department of Pediatrics, Bambino Gesù Children’s Hospital, IRCCS, Rome, Italy; ^7^ Department of Haematology/Oncology and Cell and Gene Therapy, Bambino Gesù Children’s Hospital, IRCCS, Rome, Italy; ^8^ Department of Life Sciences and Public Health, Catholic University of the Sacred Heart, Rome, Italy; ^9^ Unit of Microbiomics and Unit of Human Microbiome, Bambino Gesù Children's Hospital, IRCCS, Rome, Italy

**Keywords:** case report, cryptosporidium, CD40L immunodeficiency, chronic cryptosporidiosis, gut microbiota, protozoan parasites

## Abstract

*Cryptosporidium parvum* is a protozoan parasite and one of the leading causes of gastroenteritis in the world, primarily affecting very young children and immunocompromised patients. While infection is usually self-limiting, it can become chronic and even lethal in these vulnerable populations, in whom *Cryptosporidium* treatments are generally ineffective, due to their acting in concert with a functioning immune system. Here, we describe a case of chronic cryptosporidiosis in a European child with severe CD40L immunodeficiency infected with *Cryptosporidium parvum* of the IIa20G1 subgenotype, a lineage which has thus far only ever been described in the Middle East. After years of on-off treatment with conventional and non-conventional anti-parasitic drugs failed to clear parasitosis, we performed targeted metagenomics to observe the bacterial composition of the patient’s gut microbiota (GM), and to evaluate fecal microbiota transplantation (FMT) as a potential treatment option. We found that *C. parvum* infection led to significant shifts in GM bacterial composition in our patient, with consequent shifts in predicted intestinal functional signatures consistent with a state of persistent inflammation. This, combined with the patient’s poor prognosis and increasing parasitic burden despite many rounds of anti-parasitic drug treatments, made the patient a potential candidate for an experimental FMT procedure. Unfortunately, given the many comorbidities that were precipitated by the patient’s immunodeficiency and chronic *C. parvum* infection, FMT was postponed in favor of more urgently necessary liver and bone marrow transplants. Tragically, after the first liver transplant failed, the patient lost his life before undergoing FMT and a second liver transplant. With this case report, we present the first description of how cryptosporidiosis can shape the gut microbiota of a pediatric patient with severe immunodeficiency. Finally, we discuss how both our results and the current scientific literature suggest that GM modulations, either by probiotics or FMT, can become novel treatment options for chronic *Cryptosporidium* infection and its consequent complications, especially in those patients who do not respond to the currently available anti-parasitic therapies.

## 1 Introduction

*Cryptosporidium* is a genus of highly infectious opportunistic protozoan pathogens comprising over 30 species, two of which, *C. parvum* and *C. hominis*, are those that most commonly infect humans (Gerace et al., 2019; Pane and Putignani, 2022; Putignani, 2022). Human infection is primarily achieved via the ingestion of oocysts shed in the feces of infected individuals, as a result or either person-to-person or animal-to-person contact (Cacciò and Chalmers, 2016; Gerace et al., 2019; Pane and Putignani, 2022; Putignani, 2022). *Cryptosporidium* infection is more prevalent in low-income nations, where people are at a higher risk of experiencing conditions such as over-crowding, contact with infected livestock, and fecal contamination of food or water sources (Bouzid et al., 2018; Pane and Putignani, 2022). However, *Cryptosporidium* has a global distribution and has been found to be responsible for 60% of protozoan outbreaks in North America, Australia and Europe, despite the fact that it is also estimated to be grossly underdiagnosed, with up to 99% of infections going unreported in the US alone (Scallan et al., 2011; Shirley et al., 2012; Checkley et al., 2015). Most cases of *Cryptosporidium* infection are asymptomatic and self-limiting. However, infection can become chronic, debilitating and life-threatening in immunocompromised individuals, such as AIDS patients, people with primary immunodeficiencies, or people receiving immunosuppressive drugs (Shirley et al., 2012; Cacciò and Putignani, 2014; Cacciò and Chalmers, 2016; Gerace et al., 2019), resulting in over one million deaths between 1990 and 2015 (O’ Leary et al., 2021).

To date, nitazoxanide is the only FDA-approved treatment for *Cryptosporidium* infection, performing reasonably well in clinical trials with immunocompetent patients (Khan and Witola, 2023). However, multiple clinical trials have revealed that this treatment is largely ineffective in the immunocompromised, particularly in patients with exceedingly low CD40+ T-cell counts (Khan and Witola, 2023). Other drugs, such as the antibiotics paramomycin and azithromycin, have also been tested in both open and placebo-controlled clinical trials. The results of these clinical trials have been inconsistent, though on some occasions they have had moderate, short-term success in reducing parasitic burden, but rarely do they achieve full parasite clearance in the immunocompromised (Khan and Witola, 2023). At this time, there is no drug, FDA-approved or not, which can reliably treat cryptosporidiosis in the immunocompromised patients who are also at the highest risk dying from this parasitic infection.

The interconnected relationship between the gut microbiota (GM) and intestinal parasites has been gaining much attention in recent years, with studies in both animal models and human populations providing evidence for a three-way street of influence between the GM, parasites, and the immune system (Bär et al., 2015; Marzano et al., 2017; Leung et al., 2018; Ianiro et al., 2022). Not only have protozoan parasites been shown to alter the composition of the GM, there is also evidence that GM composition and modulation can influence the infective potential of protozoan pathogens (Bär et al., 2015; Marzano et al., 2017; Leung et al., 2018; Oliveira and Widmer, 2018; Oliveira et al., 2019; Charania et al., 2020; von Huth et al., 2021; Ianiro et al., 2022; Piazzesi et al., 2022). Furthermore, some studies have shown that probiotics can alleviate symptoms associated with *Cryptosporidium* infection, further highlighting the direct impact that the GM can have on parasitosis (Pickerd and Tuthill, 2004; Sindhu et al., 2014; Pane and Putignani, 2022). GM alterations in response to *Cryptosporidium* infection have been described in mice (Wang et al., 2023), livestock (Mammeri et al., 2020; Wang et al., 2022; Chen et al., 2023), and in very young children (Toro-Londono et al., 2019; Carey et al., 2021), but, to our knowledge, the effect of chronic cryptosporidiosis on the composition of the human microbiome has never been addressed in any immunocompromised patient. Here, we describe the first European case of chronic infection due to the *Cryptosporidium parvum* IIaA20G1 subgenotype in a child with primary immunodeficiency, a rare zoonotic lineage which, thus far, has only been described in a very small number of humans and livestock in Jordan, Kuwait and Iran (Sulaiman et al., 2005; Nazemalhosseini-Mojarad et al., 2011; Hijjawi et al., 2016). Furthermore, this is the first report of GM alterations in an immunocompromised human patient with chronic cryptosporidiosis.

## 2 Case description

**Figure 1:**
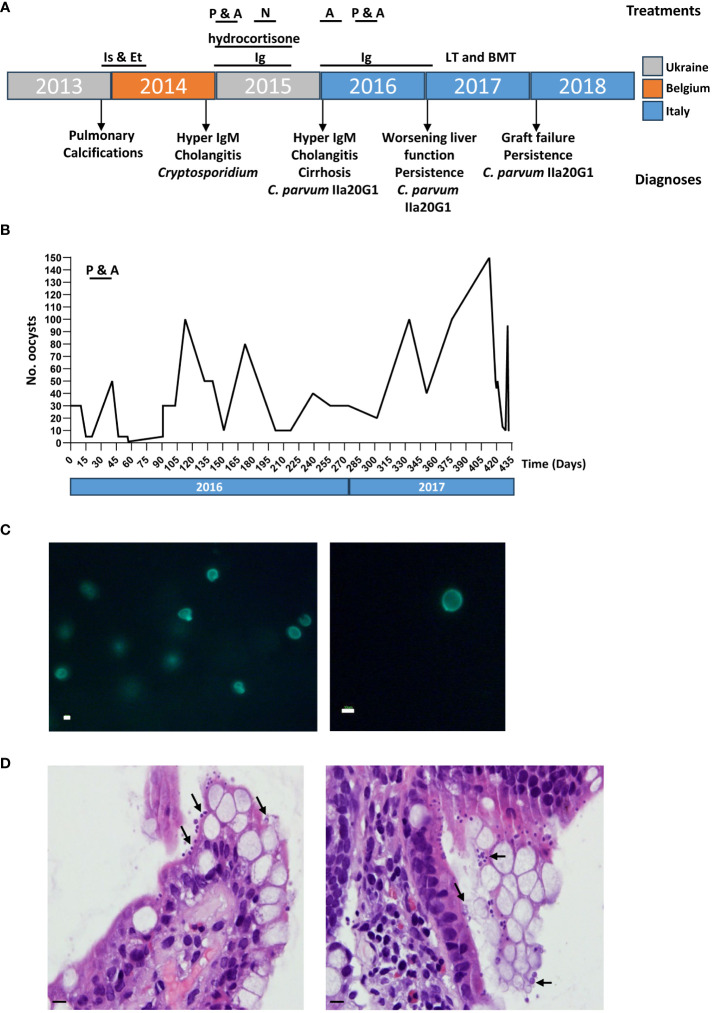
Chronic cryptosporidiosis confirmed in a patient with severe CD40L deficiency. **(A)** Timeline of patient treatment and diagnoses, color coded by country. Is & Et, Isoniazid & Ethambutol; P, Paromomycin; A, azithromycin; N, Nitazoxanide; LT, liver transplant; BMT, bone marrow transplant. **(B)** Number of oocysts detected in fecal smears from the patient over a 15-month period by Ziehl-Neelsen staining and quantitative microscopy. **(C)** MeriFluor staining of *Cryptosporidium* oocysts in fecal smears. Scale bar: 10µm. **(D)** Hemotoxylin and Eosin staining of intestinal sections show the presence of *C*. *parvum* as recognized by their proximity to the epithelial surface, being attached to the microvillus border of the enterocytes of the small bowel (indicated with black arrows). Scale bar: 50µm.

The patient was born to nonconsanguineous parents and showed normal growth and development until the 4^th^ year of life, whereupon he was hospitalized in his home country of Ukraine for persistent episodes of non-febrile diarrhea. While hospitalized the patient developed sporadic pulmonary calcifications, for which he was given three months of Isoniazid and Ethambutol treatment due to suspected tuberculosis, although the patient did not test positive for *Mycobacterium* infection. After treatment, the patient suffered from rapid weight loss, asthenia, leukocytosis and eosinophilia. An abdominal ultrasound and a liver needle biopsy confirmed the presence of liver fibrosis and cirrhosis, for which he was transferred to a Belgian hospital for further treatment. The hospital confirmed hypogammaglobulinemia, for which he was given monthly treatments with 500mg/kg Ig (**Figure 1A**). He also presented with hyporegenerative anemia, eosinophilia, elevated gamma-glutamyl transferase (GGT; 670 UI/L), bile acids (104 umol/L) total bilirubin (1.4 mg/dl) and lactate dehydrogenase (LDH; 539 UI/L), as well as hypertransaminasemia (ALT 133, AST 147 UI/L), hypercholesterolemia (341 mg%, LDL 281 mg%), hypertriglyceridemia (224 mg%) and hypocalcemia. Liver function was normal, but abdominal ultrasound and a liver needle biopsy revealed sclerosing cholangitis. Colonoscopy and esophagogastroduodenoscopy further uncovered evidence of erosive gastritis and the presence of *Cryptosporidium* spp. In addition to 500mg/kg Ig for the hypogammaglobulinemia the patient was treated with hydrocortisone for sclerosing cholangitis (**Figure 1A**), after which he returned to his home country. For his *Cryptosporidium* infection, the patient was initially treated with paromomycin and azithromycin. When this treatment failed, the patient also underwent a second round of treatment with nitazoxanide (**Figure 1A**).

For two months the patient was recovering and gaining some weight, but then had a relapse of weight loss and diarrhea. He was hospitalized again in Ukraine, where he suspended all ongoing treatments, at which point our hospital was contacted for a follow-up.

## 3 Diagnostic assessment

### 3.1 Confirmation of chronic cryptosporidiosis

At age six, the patient was transferred to the Bambino Gesù Pediatric Hospital in Rome, Italy, following his hospitalization for chronic diarrhea and rapid weight loss in his home country of Ukraine. Diagnostic tests revealed a further deterioration of hepatic function, with worsening sclerosing cholangitis and cirrhosis. Flow cytometry (O’Gorman et al., 1997) confirmed hyper IgM-syndrome confirmed by the lack of expression of CD40L, while parasitology exams, performed on stool by Ziehl–Neelsen and Merifluor^®^ staining (Del Chierico et al., 2011; Del Chierico et al., 2018), confirmed the ongoing infection by *Cryptosporidium* infection (**Figures 1B, C**). Histology on intestinal biopsies showed an overcrowding of *Cryptosporidium* staining present on the surface of the epithelium (**Figure 1D**). Subsequent DNA genotyping (Del Chierico et al., 2011) identified the infectious agent to be *Cryptosporidium parvum* belonging to the IIaA20G1 subgenotype, a zoonotic lineage described in only a few cases of human and ruminant animal infection in the Middle East (Sulaiman et al., 2005; Nazemalhosseini-Mojarad et al., 2011; Hijjawi et al., 2016). As the persistence of *C. parvum* infection put the patient at extremely high risk of rejection of a liver transplant, we decided to attempt to treat the infection once again and delay the liver transplant as much as possible. The patient was treated, first with azithromycin alone, and then with a combination of paromomycin and azithromycin (**Figure 1A**). However, while oocyst counts initially dipped in response to treatment with the two antibiotics, the infection recovered and the patient soon began suffering an exceedingly high parasitic burden (**Figure 1B**).

### 3.2 Modulation of the GM bacterial community following chronic cryptosporidiosis

After 15 months of confirmed, chronic cryptosporidiosis persisting even after four rounds of conventional and non-conventional treatment (**Figures 1A, B**), we performed an in-depth analysis of the patient’s GM composition in order to evaluate the patient’s eligibility for fecal microbiota transplantation (FMT). The patient’s GM profile was assessed by performing 16S rRNA sequencing on four stool samples collected over six days, and the results were compared to five age- and sex- matched stool samples from healthy controls (CTRLs) present in the Biobanking and Bio-Molecular Research Infrastructure (BBMRI) Biobank of the Human Microbiome Unit of the Bambino Gesù Pediatric Hospital.

**Figure 2:**
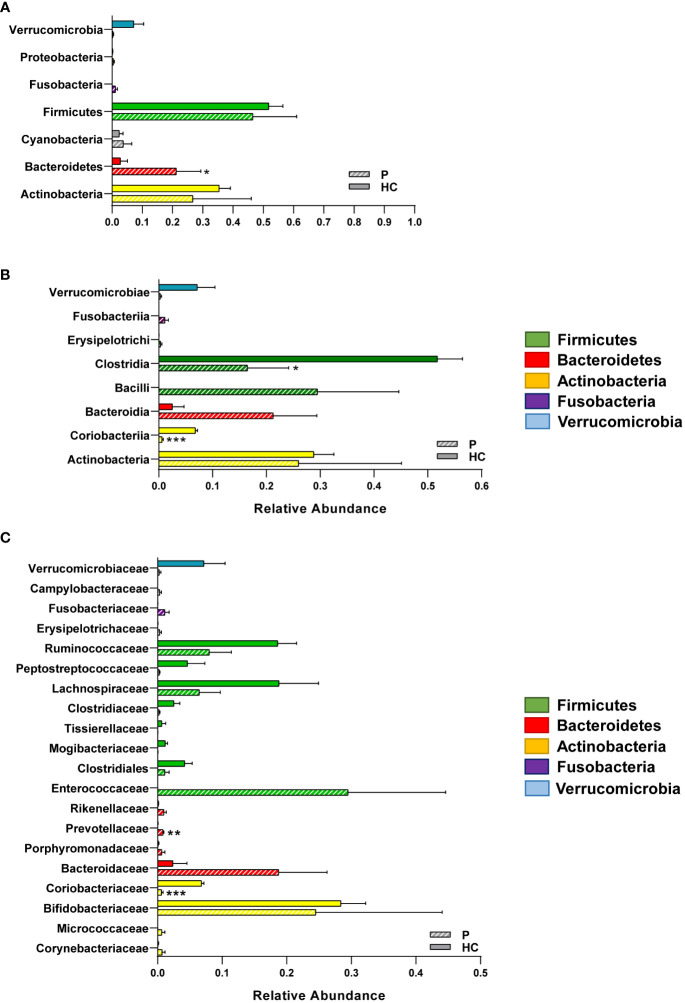
Relative abundances of the different bacterial taxa in the GM of the patient and of healthy CTRLs. Bar charts of the relative abundances of selected **(A)** phyla, **(B)** classes and **(C)** families identified in the patient and in age- and sex-matched CTRLs. Data color-coded by phylum. Data represented as mean ± S.E.M, Mann Whitney U-test, *p<0.05, **p<0.01, ***p<0.001.

While the sample sizes were too small to produce statistically significant differences in alpha and beta diversity (data not shown), we found that the patient had significant shifts in bacterial taxa at every taxonomical level consistent with a dysbiotic state, likely in response to the persistence of *C. parvum* within the intestinal niche. Specifically, the patient had reduced relative abundances of the phylum Verrucomicrobia and expanded populations of Actinobacteria and Fusobacteria, though the only phylum that was significantly increased in the patient was Bacteroidetes (**Figure 2A**). Within the Actinobacteria phylum, the Coriobacteriia class and consequently the Coriobacteriaceae family, were significantly reduced in the patient compared to CTRLs (**Figures 2B, C**). Within the Firmicutes phylum, on the other hand, the Clostridia class was significantly decreased, while Bacilli were increased in the patient compared with CTRLs (**Figure 2B**). Within the Bacteroidetes phylum, the Prevotellaceae and the Bacteroidaceae families were also increased in the patient compared to CTRLs, which is indicative of a state of intestinal inflammation (**Figure 2C**).

**Figure 3:**
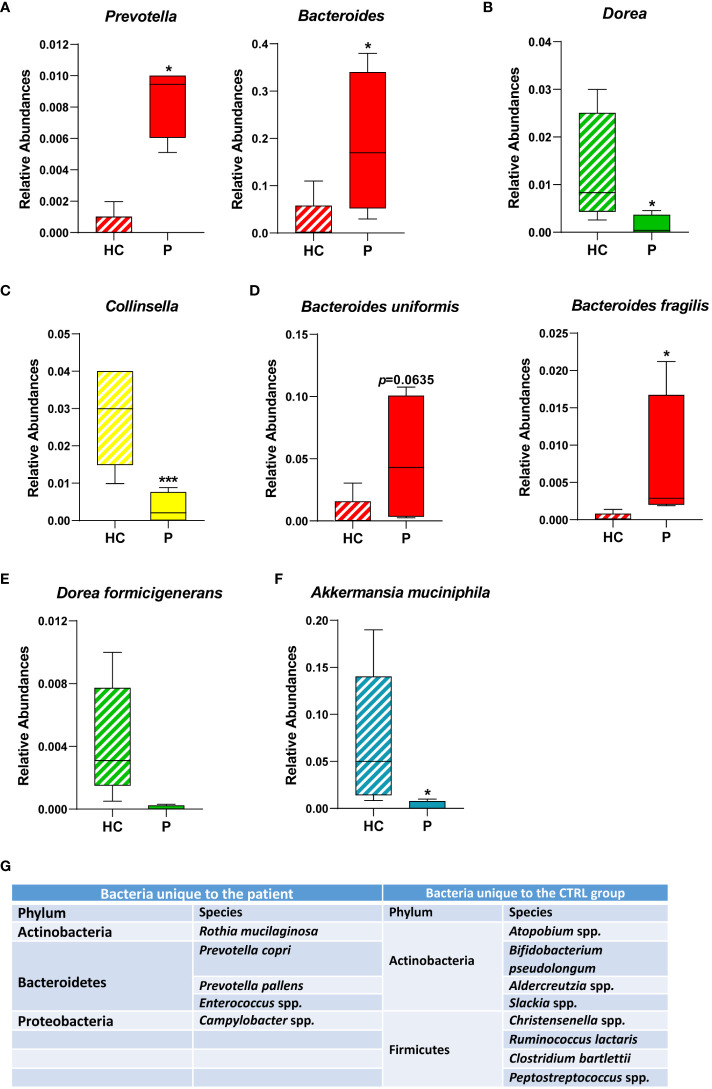
Relative abundances of bacterial genera and species identified in the GM of the patient and of healthy CTRLs. Box plots of the relative abundances of selected bacterial genera **(A-C)** and species identified **(D-F)** in the patient and in age- and sex-matched CTRLs. Data color-coded by phylum. Data represented as mean ± S.E.M, Mann Whitney-U test, *p<0.05, ***p<0.001. **(G)** Table of bacterial genera/species found exclusively in either the patient or in the healthy CTRL samples. Bacteria that were not classified beyond the family level, and those that were not identified in at least three samples were not included.

Specific genera (**Figures 3A-C**) and species (**Figures 3D-F**) commonly associated with inflammation and disease were also significantly altered in our patient, such as the *Prevotella* and *Bacteroides* genera (**Figure 3D**). Conversely, in the Firmicutes phylum, the *Dorea* genus (**Figure 3B**) and specifically the species *Dorea formicigenerans* (**Figure 3E**), were both significantly reduced in the patient compared with CTRLs, as was the genus *Collinsella* (**Figure 3C**) and the famously anti-inflammatory *Akkermansia municiphila* (**Figure 3F**) (Zheng et al., 2022).

Furthermore, specific bacterial genera and species were unique to either the patient or the CTRL groups (**Figure 3G**). Consistent with an overall increase in the genus, two species of *Prevotella*, namely *Prevotella copri* and *Prevotella pallens*, were identified exclusively in the patient samples (**Figure 3G**), the former also being consistent with reports on protozoan-induced diarrheal disease (Gilchrist et al., 2016). *Campylobacter* and *Enterococcus* were also detected exclusively in the patient, which are both genera that comprise opportunistic pathogens associated with gastroenteritis and intestinal inflammation (Casey et al., 2017). Conversely, anti-inflammatory bacteria such as *Bifidobacterim pseudolongum* and *Adlercreutzia* spp. were also depleted in the patient (**Figure 3G**). Taken together, these results are consistent with a state of sustained intestinal inflammation in response to chronic infection from *Cryptosporidium parvum*.

### 3.3 Inferred functional profile of the GM in response to cryptosporidiosis

**Figure 4:**
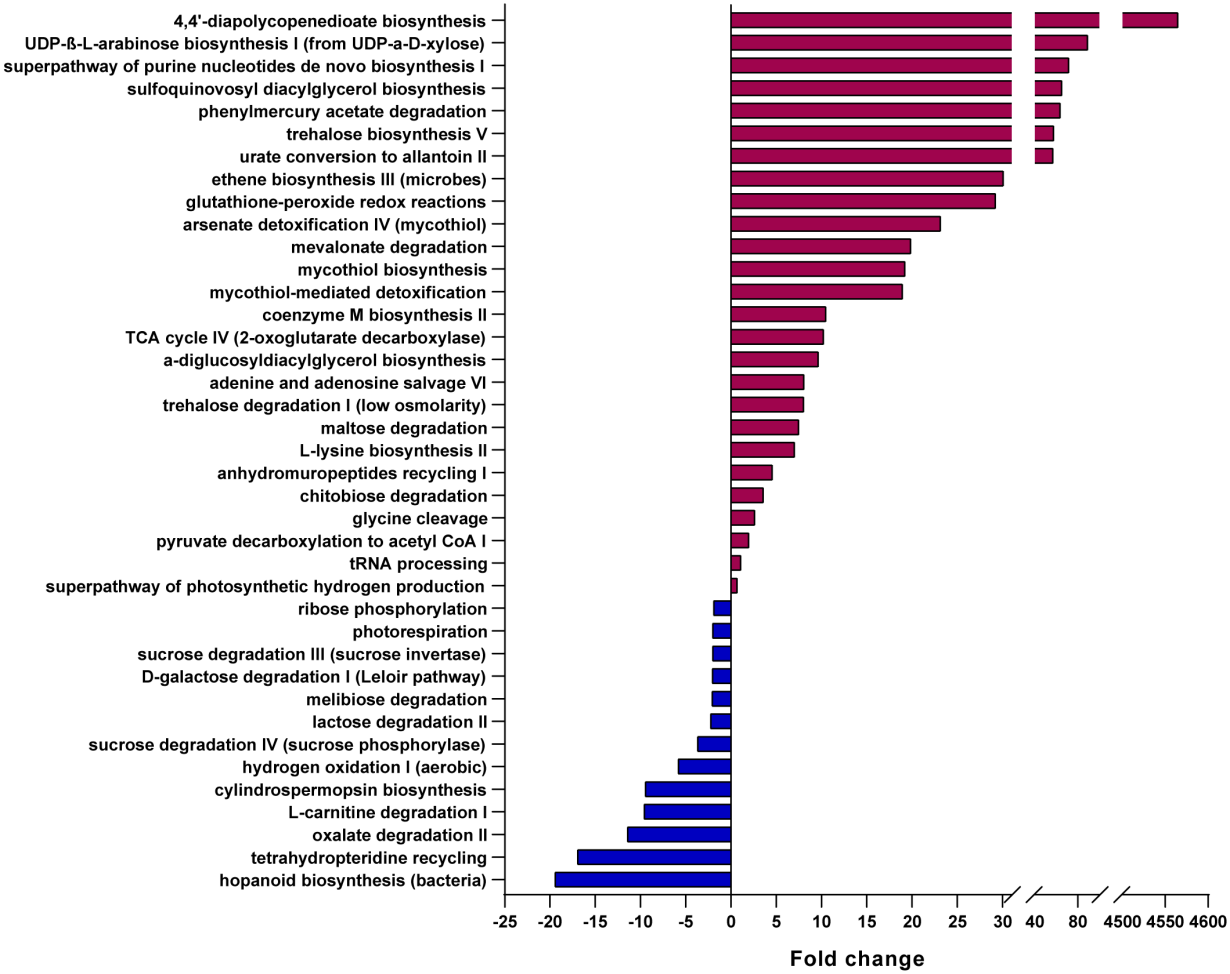
Predicted metabolic pathways overrepresented in the patient and in healthy CTRLs. Bar chart of the pathways predicted to be most significantly overrepresented in either the patient (burgundy) or healthy CTRLs (blue), using PICRUSt2, EC Database, MetaCyc Pathway Database and a confidence cutoff of 1.

Based on these microbial GM maps, we built inferred functional profiles of the patient’s GM compared to CTRLs (**Figure 4**). We found that 26 pathways were significantly overrepresented in the patient, while 13 were significantly overrepresented in the CTRL group (**Figure 4**). Of the pathways that were significantly enriched in the patient, there were many involved in carbohydrate catabolism, including the degradation of chitobiose, maltose, and trehalose (**Figure 4**). Interestingly, an increase in trehalose degradation is consistent with a previously published metabolomics study on *Cryptosporidium*-positive fecal samples (Ng et al., 2012). Furthermore, different pathways involving sugar degradation, such as lactose, D-galactose, sucrose, and ribose degradation, were found to be significantly enriched in the CTLRs, and thus are underrepresented in the patient (**Figure 4**). Consistently, the same metabolomics study on human fecal samples found that, while trehalose was depleted in *Cryptosporidium*-positive stool, sucrose was increased in these samples (Ng et al., 2012). Given that these pathways are inferred from the patient’s intestinal bacterial community profile, these results strongly suggest that the differences in carbohydrates previously found between infected and uninfected individuals, originated from the GM.

Similarly, pathways which feed into the TCA cycle, including mevalonate degradation and glycine cleavage, were also enriched in the patient compared to CTRLs, which is also consistent with an increase in TCA metabolic intermediates found in stool samples from infected patients (Ng et al., 2012). Furthermore, an increase in TCA intermediates has been described in many pathological contexts, including necrotizing enterocolitis (Du et al., 2023) and colorectal cancer (Telleria et al., 2022). Finally, we found an enrichment in purine nucleotide salvage pathways, including the superpathway of purine nucleotides, adenine and adenosine salvage, UDP-β-L-arabinose biosynthesis I (from UDP-α-D-xylose), and urate conversion to allantoin II (**Figure 4**). Purines from the GM are important sources of energy for the healing and maintenance of the intestinal mucosa (Lee et al., 2020), and thus their enrichment in the patient is consistent with the presence of intestinal lesions and distress.

### 3.4 Clinical outcome

Taken together, the ecological and inferred metabolic profile of the patient’s GM revealed a dysbiotic state, consistent with a case of persistent intestinal inflammation, distress, and consistent with other reported cases of protozoan infection. These results made the patient potentially eligible for an experimental fecal microbiota transplant (FMT), especially given the failure of all anti-parasitic treatments, his poor prognosis, the lack of other approved therapeutic options, and given the fact that his first liver transplantation failed, most likely due to his chronic cryptosporidiosis (**Figure 1A**). The patient was then evaluated for both FMT and a second liver transplant, which he unfortunately never received, after he finally succumbed to his disease before they could take place.

## 4 Discussion

We present a case report of a child with chronic cryptosporidiosis as a result of CD40L deficiency and hyper-IgM syndrome. Studies in mouse models of CD40-CD40L immunodeficiency indicate that CD40L is necessary to clear *C. parvum* infection, consistent with the findings that people affected with this disorder are at high risk of developing chronic cryptosporidiosis (Cosyns et al., 1998; Subauste, 2009). CD40L is expressed on activated CD4+ T cells, and is required for many critical steps of cell-mediated immunity, such as B cell proliferation, isotype switching, Ig secretion, and memory cell production (Mir, 2015; Pai and Notarangelo, 2018). The precise molecular mechanism of how CD40L expression mediated parasite clearance remains to be fully elucidated, and likely relies on multiple mechanisms. Upregulation of CD40L is crucial for T-cell activation, by binding to CD40 on macrophages, can stimulate IL-12 production and the upregulation of costimulatory molecules. An inability to express CD40L interfere with T-cell activation and hence with the clonal expansion of antigen-specific T cells, which is required for an effective cell-mediated immunity. Studies in both mice and human cell lines indicate possible direct antimicrobial activity of CD40 activation, possibly by inducing apoptosis in infected cells (Hayward et al., 1997; Hayward et al., 2001; Subauste, 2009). Furthermore, Secretory IgA (SIgA) in the intestinal mucosa, whose production is also dependent on CD40L, may contribute to the host’s protection against cellular invasion by *Cryptosporidium* (Dann et al., 2000; Pietrzak et al., 2020).

We present the first European case of *C. parvum* infection of the IIaA20G1 subgenotype which we speculate, given the patient’s medical history, was acquired in his home country of Ukraine. This particular zoonotic lineage of *Cryptosporidium* has, currently, only been described in the Middle East, where it has been described in a small number of humans, sheep, and goat infections (Nazemalhosseini-Mojarad et al., 2011; Hijjawi et al., 2016; Majeed et al., 2018). Given the zoonotic capabilities of this lineage and the fact that the patient was already infected when he arrived to our hospital, we cannot deduce whether infection was caused by person-to-person or animal-to-person contact. Furthermore, the apparent relative rarity of this particular subgenotype makes it impossible for us to know how much of the severity of this case was due to the child’s immunodeficiency and clinical history, and how much was due to the infectious potential of the IIaA20G1 lineage. Future studies, which combine epidemiological tracing with clinical data on infectivity and disease severity, could shed more light on which subgenotypes, and consequently which reservoirs, pose the biggest threat to vulnerable populations.

Here, we present the first bacterial community profile of an immunocompromised child with chronic cryptosporidiosis. We found that, after over a year of parasitic infection and failure of specific treatment, the gut bacterial community composition was significantly altered. We observed a significant increase in the phylum Bacteroidetes, which was largely due to increased abundances of the *Prevotella* and *Bacteroides.* An increase in the *Prevotella* genus, which has previously been connected to inflammatory disorders (Larsen, 2017), was consistent with reported microbial signatures associated with infections triggered by other intestinal protozoans, such as *Giardia* (Toro-Londono et al., 2019; Berry et al., 2020; Mejia et al., 2020), *Blastocystis* (Andersen et al., 2015; Pane et al., 2021) and *Entamoeba histolytica* (Gilchrist et al., 2016), as well as microbiome studies of mice infected with *Cryptosporidium* (Wang et al., 2023). These results suggest that an abundance of *Prevotella* spp. is a common signature in intestinal protozoan infection. Furthermore, we found other opportunistic pathogenic bacterial genera, such as *Campylobacter* and *Enterococcus* which, taken together, paint a picture of sustained intestinal inflammation in our patient in response to chronic *Cryptosporidium* infection.

Consistently, we found a significant reduction in bacterial genera and species that are known to possess anti-inflammatory properties, such as *A. muciniphila*, and *B. pseudolongum. A. muciniphila* has been positively correlated with childhood growth and negatively correlated with gastroenteritis in children living in areas with a high prevalence of enteric pathogens (George et al., 2023). Furthermore, *A. muciniphila* abundance is negatively correlated with a wide range of diseases and positively correlated with favorable outcomes in cancer patients, making this bacterial species the focus of novel, “next generation” probiotic studies (Yi et al., 2018; Ponziani et al., 2019; Zhai et al., 2019; Zhang et al., 2019). *B. pseudolongum* supplementation, on the other hand, has been found to be protective in mouse models of colitis (Tatsuoka et al., 2023), hyperlipidemia (Zhang et al., 2021), and organ transplant recipients (Mongodin et al., 2017), although its effects in humans, positive or otherwise, remains to be fully elucidated.

There is already some evidence, from both animal models and from human studies, to suggest that modulations of the GM are a viable option in the treatment of cryptosporidiosis (Pickerd and Tuthill, 2004; Pane and Putignani, 2022). One clinical study on very young children showed that supplementing treatment with the probiotic *Lactobacillus rhamnosus GG* improved intestinal function in children with cryptosporidial diarrhea (Sindhu et al., 2014). In one intriguing case, *Cryptosporidium* oocysts were completely cleared following a four week course of both *Lactobacillus rhamnosus GG* and *Lactobacillus casei* (Pickerd and Tuthill, 2004). In a mouse model of immunosuppressed, *Cryptosporidium*-infected mice, the probiotic *Enterococcus faecalis* CECT 7121 was able to interfere with the parasite and alleviate some of the negative effects of infection (Del Coco et al., 2016). Consistently, another study on immunosuppressed mice showed that prophylactic treatment with *Lactobacillus reuteri* made it possible for mice to clear parasitic infection, while untreated mice developed persistent cryptosporidiosis (Alak et al., 1997). These findings are particularly important because they suggest that, while the GM can often act on infectious pathogens indirectly through the immune system, these mice did not need a functioning immune system to benefit from probiotic administration, indicating the GM can act on parasitosis in a more direct manner. Consistently, *in vitro* studies have found that GM-derived metabolites, such as indoles, can reduce host mitochondrial function and depolarize the *Cryptosporidium* mitosome, which in turn compromises *Cryptosporidium* growth and survival (Funkhouser-Jones et al., 2023). It is also important to note that the current available treatments for cryptosporidiosis are not efficient in the immunocompromised because they too act in concert with the immune system. Therefore, taken together, these findings strongly suggest that modulations of the GM can be beneficial for *Cryptosporidium*-infected patients even without a functioning immune system. However, despite these promising findings, to date there have been no reports on the effects of GM modulations, either by probiotics or FMT, on immunocompromised human patients with chronic cryptosporidiosis. With this case report, we believe that our results further support the hypothesis that GM modulation is a field which requires more attention and study, and which can develop into an attractive alternative therapeutic strategy for immunocompromised patients who are resistant to anti-*Cryptosporidium* treatments.

## Data availability statement

Datasets analyzed in this study are publicly available on the NCBI Sequence Read Archive (SRA) under the accession number PRJNA1013917.

## Ethics statement

Informed written consent for the publication of this case report was obtained from the patient’s legal guardian. The use of healthy subjects in this study was approved by the Ethical Committee of the Bambino Gesù Children's Hospital, IRCCS (protocol No. 1113_OPBG_2016) and was conducted in accordance with the Principles of Good Clinical Practice and the Declaration of Helsinki. Written informed consent was obtained from the legal guardians of healthy subjects. Written informed consent was obtained from the participant/patient(s) for the publication of this case report.

## Author contributions

AP: Formal Analysis, Validation, Visualization, Writing – original draft, Writing – review & editing. SP: Formal Analysis, Investigation, Validation, Writing – review & editing. AR: Investigation, Validation, Writing – review & editing. FDC: Investigation, Writing – review & editing. PF: Validation, Writing – review & editing. NC: Resources, Writing – review & editing. PR: Resources, Validation, Writing – review & editing. FL: Resources, Supervision, Writing – review & editing. PP: Conceptualization, Resources, Supervision, Writing – review & editing. LP: Conceptualization, Funding acquisition, Project administration, Supervision, Writing – original draft, Writing – review & editing.
